# Distributed Adaptive Optimization Algorithm for High-Order Nonlinear Multi-Agent Stochastic Systems with Lévy Noise

**DOI:** 10.3390/e26100834

**Published:** 2024-09-30

**Authors:** Hui Yang, Qing Sun, Jiaxin Yuan

**Affiliations:** College of Air Transportation, Shanghai University of Engineering Science, Shanghai 201620, China; 08130002@sues.edu.cn (H.Y.); sq890622@163.com (Q.S.)

**Keywords:** stochastic multi-agent systems, adaptive backstepping control, command filter, distributed optimization problem, Lévy noise

## Abstract

An adaptive neural network output-feedback control strategy is proposed in this paper for the distributed optimization problem (DOP) of high-order nonlinear stochastic multi-agent systems (MASs) driven by Lévy noise. On the basis of the penalty-function method, the consensus constraint is removed and the global objective function (GOF) is reconstructed. The stability of the system is analyzed by combining the generalized Itô’s formula with the Lyapunov function method. Moreover, the command filtering mechanism is introduced to solve the “complexity explosion” problem in the process of designing virtual controller, and the filter errors are compensated by introducing compensating signals. The proposed algorithm has been proved that the outputs of all agents converge to the optimal solution of the DOP with bounded errors. The simulation results demonstrate the effectiveness of the proposed approach.

## 1. Introduction

Research of stochastic systems has gained considerable attention in recent years. For example, Liang [1] constructed a common output feedback controller, independent of the switching signal, using the backstepping method to solve the global output feedback probability stability problem for a class of switching random nonlinear systems under arbitrary switching. Fang [2] explored a novel adaptive optimal control strategy for a class of sophisticated discrete-time nonlinear Markov jump systems via Takagi–Sugeno fuzzy models and reinforcement learning techniques. Furthermore, the usual sense stochastic noise is just driven by continuous Brownian motion [3,4], which may be a description of continuous stochastic volatility.

However, numerous types of discontinuous noise exist in many physical systems, for instance, random faults, abrupt changes, and sudden disruptions [5]. Furthermore, yet, there is a distinct type of noise, namely “Lévy noise”, which can characterize both Brownian motion and Poisson jump processes [6]. To date, there have been advancements in filed of control the stochastic systems with Lévy noise [7,8,9,10], but there has been no research of the consensus problem for MASs with Lévy noise. Furthermore, consensus control algorithms for MASs with stochastic noise which is not Lévy noise have developed rapidly and a substantial body of literature has been produced. For example, Refs. [11,12,13] discussed the stochastic linear MASs. Ref. [11] developed a two-step algorithm for each agent in order to dynamically estimate the states of its neighbors. Controllers based on the error between the estimated states of the neighbors and the complete state of the agent were designed by [12], aimed at resolving the limited consensus problem in continuous-time linear MASs with additive systems and communication noise. Ref. [13] introduced the innovative notion of the sub-accessibility of the sliding motion approaching a particular sliding surface for generic MASs which are driven by Brownian motion. References [14,15,16,17] discussed the stochastic nonlinear MASs. In detail, Ref. [14] investigated the consensus tracking problem for the MASs which have outputs, partial state constraints, and saturated inputs, where the unmodeled dynamics are evaluated by the use of RBFNN. In [15], the authors explored a class of fuzzy adaptive leader–follower tracking control problems for MASs with stochastic noise which have unknown dead-zone inputs, where the stochastic disturbances and uncertain functions of the MAS are approximated by introducing a fuzzy logic system. Furthermore, to ensure that all agents reach a consensus within a limited time frame, Ref. [16] has developed a distributed control algorithm based on stochastic stability theorems in finite-time and has integrated a power integrator technique. To achieve the consensus of stochastic nonlinear MASs under directional communication topology, Ref. [17] put forward a distributed adaptive fuzzy control scheme, which employs the integral mean theorem and the approximation properties of fuzzy logic systems.

In the above works, the research of MASs only focus on reaching the basic consensus behavior. However, distributed optimization is commonly preferred for real applications. The DOP is an expansion of the MASs consensus problem and refers to addressing the DOP on the basis of consensus. The main goal of DOP is to achieve the minimization of the GOF, which is the summary of all the local objective functions [18]. The result is that all of the agents are cooperating in trying to achieve the optimal value of the GOF. One of the key goals of DOP for MASs is the design of adequately distributed controllers [19], so that all MASs can converge cooperatively under a certain communication topology, and achieve the optimal solution of distributed optimization after convergence.

There have been several work applications to the study of designing distributed optimization algorithms based on first-order MASs and second-order MASs for solving the DOP [20,21,22,23,24]. Based on event-triggered strategies, Ref. [20] designed a distributed optimization algorithm to solve the DOP of continuous-time first-order MASs with external disturbances and discrete communication. Ref. [23] proposed an improved distributed continuous-time algorithm to design an event-triggered algorithm for the solution of a generalized DOP. However, numerous realistic systems, like manipulators and helicopters, cannot be represented by these low-order dynamics. Therefore, the DOP of high-order nonlinear MASs has attracted the attention of some scholars. For example, Ref. [25] constructed a bounded local control law for achieving global optimal consensus in MASs under the assumption that all agents reach an agreement on the condition of minimizing the summary of all agents’ objective function. An adaptive Lyapunov-based backstepping method was proposed by [26] to decompose the DOP of high-order MASs into an optimization or control problem for solving multiple first-order subsystems. Ref. [27] looked into the subject of the optimal output consensus and proposed an embedded control system which using an optimal signal generation technique. Ref. [28] investigated a distributed optimization algorithm of bipartite containment control for high-order MASs with state constraints. However, DOP research for high-order nonlinear stochastic systems has not yet been conducted.

Motivated by the above analysis, an adaptive NNs backstepping controller which is based on the command filter is developed in this article to solve the DOP of high-order nonlinear MASs which contain the Lévy stochastic noise. The significant contributions of this work in comparison to previous research are listed below.

(1) In contrast to [29,30,31], which only applied the adaptive control method based on observer to address the consensus problem, we focus on resolving the DOP for MASs with unmeasurable states. A distributed optimal adaptive controller is introduced to solve this problem, which utilizes the penalty function and the negative gradient. The objective of this controller is to ensure that the outputs of all the agents will progressively arrive at the optimal value of the GOF.
(2) All of Refs. [32,33,34] only solved the low-order MASs consensus problem with stochastic noise; in this article, a distributed optimal backstepping controller is proposed to solve the DOP for high-order MASs with unmeasurable states and Lévy noise.
(3) Different from our study, Refs. [25,27,35,36,37] did not combine neural networks (NNs), observers, and command filtering to solve the DOP for MASs with Lévy noise. NNs are used for approximation of unknown nonlinear functions and stochastic noise, and observers are used to obtain unmeasured states. We combine the command-filtered control technology with the error compensation technology to solve the problem of “explosion of complexity” and eliminate the effect of filtering errors.

## 2. Preliminaries

### 2.1. Graph Theory

Consider the MASs involving *n* agents, we take an undirected graph $Q=\left(M,Z,\bar{A}\right)$ to represent the relationship between agents, where $M=\left\{m_{1},\ldots ,m_{N}\right\}$ is a node set, $Z=\left\{\left(m_{i},m_{j}\right)\right\}\in M\times M$ stands for the edge set and $\bar{A}=\left\{a_{ij}\right\}\in R^{N\times N}$ is the adjacency matrix. An edge $\left(m_{i},m_{j}\right)\notin Z$, if and only if $a_{ij}=0$. Denote $N_{i}=\left\{j\left|\left(m_{i},m_{j}\right)\in Z\right.\right\}$ as the neighbor set of node ß and the matrix $D=diag\left(d_{1},\ldots ,d_{N}\right)$, $d_{i}=\sum_{j\in n=N_{i}}a_{ij}$ as the degree matrix. The Laplacian matrix $L=D-\bar{A}$.

### 2.2. Multi-Agent System

Take the MASs involving *n* agents and the dynamic for agent *i* with Lévy noise is:

(1) $$\begin{array}{c} \left\{\begin{array}{cc} dx_{i,m}\left(t\right) & =\left[x_{i,m+1}+h_{i,m}\left(X_{i,m}\right)\right]dt+F_{i,n}\left(X_{i,n}\left(t\right),t\right)dw\left(t\right)\\ & +\int_{R}G_{i,m}(X_{i,n}\left(t\right),t,\zeta )N\left(dt,d\zeta \right)\\ dx_{i,n}\left(t\right) & =\left[u_{i}\left(t\right)+h_{i,n}\left(X_{i,n}\right)\right]dt+F_{i,n}\left(X_{i,n}\left(t\right),t\right)dw\left(t\right)\\ & +\int_{R}G_{i,n}(X_{i,n}\left(t\right),t,\zeta )N\left(dt,d\zeta \right)\\ y_{i}\left(t\right) & =x_{i,1}\left(t\right) \end{array}\right. \end{array}$$

where m=1,⋯,n, $X_{i,m}={\left(x_{i,1},x_{i,2},\cdots ,x_{i,m}\right)}^{T}\in R^{m}$ are the system states vectors, $u_{i}$ is the control input of the system, $y_{i}$ represent system outputs, and $h_{i,m}\left(X_{i,m}\right)$ are unknown nonlinear functions.

Assume that $\left(\Omega ,F_{t},{\left\{F\right\}}_{t\geq 0},P\right)$ is a complete probability space. Furthermore, $w\left(t\right)$ be a one-dimensional $F_{t}$-adapted Brownian motion. $N\left(t,\zeta \right)$ is a $F_{t}$-adapted Poisson random measure defined on with intensity measure π and a compensator $\tilde{N}$. We decide that *N* is not related to *B* and ϑ is a Lévy measure with $\tilde{N}\left(dt,d\zeta \right):=N\left(dt,d\zeta \right)-\vartheta \left(d\zeta \right)dt$, where the pair $\left(B,N\right)$ is called a Lévy noise.

### 2.3. Problem Formulation

Communication topology for undirected connected, DOP is portrayed as:

(2) $$\begin{array}{c} \min_{x\in R^{N}}{£}_{i}\left(x_{i}\right),s.t.Lx=0_{N} \end{array}$$

where $x={\left[x_{1},\cdots ,x_{N}\right]}^{T}$. The approximate optimization problem is formulated using the principles of penalty function theory, which is described below:

(3) $$\begin{array}{c} \min_{x\in R^{N}}\sum_{i=1}^{N}{£}_{i}\left(x_{i}\right)+\frac{1}{2}\eta x^{T}Lx \end{array}$$

where η>0 is a constant penalty parameter and $\frac{1}{2}\eta x^{T}Lx$ is the penalty term for a violation of the consensus constraint $Lx=0_{N}$.

This article deals with DOP, the GOF $£:R^{N}\to R$ is defined as the sum of the strictly convex objective function ${£}_{i}$:

(4) $$£\left(x_{1}\right)=\sum_{i=1}^{N}{£}_{i}\left(x_{i,1}\right).$$

where $x_{1}={\left[x_{1,1}\;x_{2,1}\;\cdots \;x_{N,1}\right]}^{T}$. According to [38], $1_{N}$ is the eigenvector of the Laplacian matrix for the eigenvalue 0; when α∈R, if $x_{1}=\alpha \cdot 1_{N}$, we can get:

(5) $$Lx_{1}=0.$$

(6) $$\begin{array}{c} x_{1}^{T}Lx_{1}=0. \end{array}$$

Then, based on (4) and (6), we can define the penalty function as:

(7) $$P\left(x_{1}\right)=\sum_{i=1}^{N}{£}_{i}\left(x_{i,1}\right)+x_{1}^{T}Lx_{1}.$$

This paper intends to develop a control input $u_{i}$ in order that each agent $\lim_{t\to \infty }x_{i,1}\left(t\right)\to x_{i,1}^{*}$. Let the optimal solution $x_{1}^{*}=\left(x_{1,1}^{*},\ldots ,x_{N,1}^{*}\right)$. Define the optimal solution $x_{i,1}^{*}$ for agent *i* as:

(8) $$\left(x_{1,1}^{*},\ldots ,x_{N,1}^{*}\right)=\underset{\left(x_{1,1},\ldots ,x_{N,1}\right)}{\arg\min}P\left(x_{1}\right).$$

According (7) and (8), when the MASs receive the optimal solution $x_{1}^{*}$ we will get that all agents will achieve consensus and synchronously arrive at the optimal solution.

Then, we can develop the local objective function for agent *i* as:

(9) $$\begin{array}{c} {£}_{i}\left(x_{i,1}\right)=m_{i}x_{i,1}^{2}+\tau_{i}x_{i,1}+n_{i} \end{array}$$

where $m_{i}>0$ and $n_{i}$ and $\tau_{i}$ are constants, with 1≤i≤N.

**Remark** **1.** *From *(7)*, we can see the penalty function consists of two parts. Where $\sum_{i=1}^{N}{£}_{i}\left(x_{i,1}\right)$ is the GOF and $x_{1}^{T}Lx_{1}$ is the penalty term which can make all agents to the consensus. The purpose of this article is to construct a controller that can be satisfied to minimize the penalty function while minimizing the GOF and ensure the agents achieve consensus.*

The following lemmas are used to facilitate the calculation.

**Lemma** **1** ([10])**.** *Let $V\left(x,t\right)\in C^{2,1}\left(R^{n}\times R^{+};R\right)$, that can be continuously differentiated twice in x and once in t, and then we can design the operator LV as follows:*

(10) $$\begin{array}{cc} LV\left(x,t\right)= & V_{t}\left(x,t\right)+V_{x}\left(x,t\right)\tau \left(x,t\right)\\ \; & +\frac{1}{2}tr\left(\sigma^{T}\left(x,t\right)V_{xx}\left(x,t\right)\sigma \left(x,t\right)\right)\\ \; & +\int_{R}\left(V\left(x+\kappa \left(x,t,\zeta \right)\right)-V\left(x,t\right)\right)\vartheta \left(d\zeta \right) \end{array}$$

where

$$\begin{array}{cc} \; & V_{x}\left(x,t\right)=\left(\frac{\partial V\left(x,t\right)}{\partial x_{1}},\ldots ,\frac{\partial V\left(x,t\right)}{\partial x_{n}}\right)\\ \; & V_{xx}\left(x,t\right)={\left(\frac{\partial^{2}V\left(x,t\right)}{\partial x_{k}\partial x_{l}}\right)}_{n\times n}\\ \; & V_{t}\left(x,t\right)=\frac{\partial V\left(x,t\right)}{\partial t} \end{array}$$

and $\tau \left(x,t\right)$ represent the parameters of drift term, $\sigma \left(x,t\right)$ represent the Brownian motion term, and $\kappa \left(x,t,\zeta \right)$ for the Poisson jump term.

**Lemma** **2** ([39])**.** *The command filter is defined as:*

(11) $$\begin{array}{c} {\dot{\bar{a}}}_{i,1}=\kappa_{n}{\bar{a}}_{i,2}\\ {\dot{\bar{a}}}_{i,2}=-2\varsigma \kappa_{n}{\bar{a}}_{i,2}-\kappa_{n}\left({\bar{a}}_{i,1}-\alpha_{i}\right) \end{array}$$

*where $\varsigma \in \left(0,1\right]$ and $\kappa_{n}>0$ are the positive parameters to be designed and ${\bar{a}}_{i,1}$ and $\alpha_{i}$ express the command filter output signal and input signal.*

$${\bar{a}}_{i,1}\left(0\right)=\alpha_{i}\left(0\right),\;{\bar{a}}_{i,2}\left(0\right)=0.$$

**Lemma** **3** ([40])**.** *Any $H_{1},H_{2}\in R^{n}$, satisfied that:*

(12) $${H_{1}}^{T}H_{2}\leq \frac{\lambda^{\varpi }}{\varpi }{∥H_{1}∥}^{\varpi }+\frac{1}{\psi \lambda^{\psi }}{∥H_{2}∥}^{\psi }$$

where ϖ>1, λ>0, ψ>1, and $\left(\varpi -1\right)\left(\psi -1\right)=1$.

**Lemma** **4** ([41])**.** *A function $V\left(x,t\right)\in C^{2}$, two functions $\Upsilon_{1}$ and $\Upsilon_{2}$$\in K_{\infty }$, two positive constant ℑ and I, $t>t_{0}$, we can get that:*

(13) $$\begin{array}{c} \Upsilon_{1}\left(∥x∥\right)\leq V\left(x\right)\leq \Upsilon_{2}\left(∥x∥\right)\\ LV\left(x,t\right)\leq -ℑV\left(x,t\right)+I \end{array}$$

*for $\forall x\in R^{n}$. Thereafter, the system exists as a distinct solution and is $E\left[V\left(t\right)\right]\leq V\left(0\right)e^{-At}+B/A$ compliant. Then, all signals have probability bounds.*

**Lemma** **5.** *Under [7], the experimental solution of the *(1)* is said to be:*

*1.*  *At the origin it is almost certainly globally stable when $℘\in \left(0,1\right)$, a class $K_{\infty }$-function κ exists let $P\left\{∥X_{i,l}\left(t\right)∥<\kappa \left(∥X_{i,l}\left(t_{0}\right)∥\right)+{℘}_{0}\right\}>1-℘$, for all $t\in [t_{0},\infty )$ and $X_{i,l}\left(t_{0}\right)\in R^{n}$, ${℘}_{0}$ is a nonnegative constant.*
*2.*  *At the origin, it is almost certainly the global real $K_{\infty }$-index that is stable and $∥X_{i,l}\left(t\right)∥⩽\kappa \left(∥X_{i,l}\left(t_{0}\right)∥\right)e^{-℘\left(t-t_{0}\right)}+{℘}_{0}$, for all $X_{i,l}\left(t_{0}\right)\in R^{n}$, κ is a category $K_{\infty }$-function, ℘ is a positive constant, and ${℘}_{0}$ is a nonnegative constant.*

## 3. Main Results

### 3.1. Observer Design

Since only $y_{i}$ is measurable in the system, we need a state observer to estimate the unmeasurable state. Before designing the observer, the system is rewritten for agent *i* as:

(14) $$\begin{array}{cc} dX_{i,n} & =[A_{i}X_{i,n}+K_{i}y_{i}+\sum_{l=1}^{n}B_{i,l}\left[h_{i,l}\left(X_{i,l}\right)\right]+B_{i}u_{i}\left(t\right)y_{i}]dt\\ \; & +B_{i,l}\left\{F_{i}\left(X_{i,l}\left(t\right),t\right)dw\left(t\right)+\int_{R}G_{i}(X_{i,l}\left(t\right),t,\zeta \right)N\left(dt,d\zeta \right)\}\\ y_{i}\left(t\right) & =C_{i}X_{i,n} \end{array}$$

where $A_{i}=\left[\begin{array}{cccc} -\iota_{i,l} & & & \\ \vdots & & I_{n-1} & \\ -\iota_{i,n} & 0 & \cdots & 0 \end{array}\right]$, $K_{i}=\left[\begin{array}{c} \iota_{i,1}\\ \vdots \\ \iota_{i,n} \end{array}\right]$, $B_{i}=\left[\begin{array}{c} 0\\ \vdots \\ 1 \end{array}\right]$, $B_{i,l}={\left[0\cdots 1\cdots 0\right]}^{T}$, $C_{i}=\left[1\;0\cdots 0\right]$.

There subsists a positive matrix $P_{i}^{T}=P_{i}$ which satisfies $A_{i}^{T}P_{i}+P_{i}A_{i}=-2Q_{i}$ with respect to the given positive matrix $Q_{i}^{T}=Q_{i}$.

Since the nonlinear functions $h_{i,l}\left(X_{i,l}\right)$ are unknown, we can take a lemma as follows.

**Lemma** **6** ([42])**.** *Being an excellent means of approximating continuous functions, the paper makes use of RBFNN to compensate for the nonlinear functions $h_{i,l}$, i=1,⋯,n. The unknown function may be displayed as follows:*

(15) $$h_{i,l}\left(X_{i,l}|\psi_{i,l}\right)=\psi_{i,l}^{T}\varphi_{i,l}\left(X_{i,l}\right)$$

*where $X_{i,l}$ is the input vector, 1≤i≤n, $\varphi_{i,l}\left(X_{i,l}\right)$ is the Gaussian basis function vector, and $\psi_{i,l}$ is the ideal constant vector.*

The state of the MASs (1) are assumed not to be provided in this article. Therefore, agent *i*’s states must be estimated by an observer. Under these condition, we can define the observer:

(16) $$\begin{array}{cc} {\dot{\hat{X}}}_{i,n} & =A_{i}{\hat{X}}_{i,n}+K_{i}y_{i}+\sum_{l=1}^{n}B_{i,l}\left[{\hat{h}}_{i,l}\left({\hat{X}}_{i,l}|\psi_{i,l}\right)\right]+B_{i}u_{i}\left(t\right)\\ \hat{y_{i}} & =C_{i}{\hat{X}}_{i,n} \end{array}$$

where ${\hat{X}}_{i,l}$ represents the estimated values of $X_{i,l}$.

According to (14) and (16), we will get:

(17) $$\begin{array}{cc} \; & de_{i}\left(t\right)\\ \; & =[A_{i}e_{i}+\sum_{l=1}^{n}B_{i,l}\left[h_{i,l}\left({\hat{X}}_{i,l}\right)-{\hat{h}}_{i,l}\left({\hat{X}}_{i,l}\left|\psi_{i,l}\right.\right)+\Delta h_{i,l}\right]]dt\\ \; & +B_{i,l}\left(F_{i}\left(X_{i,l}\left(t\right),t\right)dw\left(t\right)+\int_{R}G_{i}(X_{i,l}\left(t\right),t,\zeta \right)N\left(dt,d\zeta \right)) \end{array}$$

where $\Delta h_{i,l}=h_{i,l}\left(X_{i,l}\right)-h_{i,l}\left({\hat{X}}_{i,l}\right)$ and $e_{i}=X_{i,n}-{\hat{X}}_{i,n}$ is the error in observing the state of the system (1).

According to lemma 6, we can get that:

(18) $${\hat{h}}_{i,l}\left({\hat{X}}_{i,l}\left|\psi_{i,l}\right.\right)=\psi_{i,l}^{T}\varphi_{i,l}\left({\hat{X}}_{i,l}\right).$$

The optimal parameter vectors are defined as:

(19) $$\psi_{i,l}^{*}=\arg\min_{\theta_{i,l}\in \Omega_{i,l}}\left[\underset{{\hat{X}}_{i,l}\in U_{i,l}}{\sup}\left|{\hat{h}}_{i,l}\left({\hat{X}}_{i,l}\left|\psi_{i,l}\right.\right)-h_{i,l}\left({\hat{X}}_{i,l}\right)\right|\right]$$

Define the parameter estimation error ${\tilde{\psi }}_{i,l}$ and parameter estimation $\varepsilon_{i,l}$ as:

(20) $$\begin{array}{cc} {\tilde{\psi }}_{i,l} & =\psi_{i,l}^{*}-\psi_{i,l},l=1,2,\ldots ,n.\\ \varepsilon_{i,l} & =h_{i,l}\left({\hat{X}}_{i,l}\right)-{\hat{h}}_{i,l}\left({\hat{X}}_{i,l}\left|\theta_{i,l}^{*}\right.\right) \end{array}$$

**Assumption** **1** ([43,44])**.** *The errors of the optimal approximation remain bounded and there are positive constants $\varepsilon_{i0}$ satisfying $\left|\varepsilon_{i,l}\right|\leq \varepsilon_{i0}$.*

**Assumption** **2.** *There exist some known constants $\gamma_{i}$ which are related as follows:*

(21) $$\left|h_{i,l}\left(X_{i,l}\right)-h_{i,l}\left({\hat{X}}_{i,l}\right)\right|\leq \gamma_{i,l}∥X_{i,l}-{\hat{X}}_{i,l}∥.$$

By Equations (16) and (17), we have:

(22) $$\begin{array}{cc} de_{i}\left(t\right) & =[A_{i}e_{i}+\Delta h_{i}+\varepsilon_{i}+\sum_{l=1}^{n}B_{i,l}\left[{\tilde{\psi }}_{i,l}^{T}\varphi_{i,l}\left({\hat{X}}_{i,l}\right)\right]]dt\\ \; & +B_{i,l}(F_{i}\left(X_{i,l}\left(t\right),t\right)dw\left(t\right)\\ \; & +\int_{R}G_{i}(X_{i,l}\left(t\right),t,\zeta )N\left(dt,d\zeta \right)) \end{array}$$

where $\varepsilon_{i}={\left[\varepsilon_{i,1},\ldots ,\varepsilon_{i,n}\right]}^{T}$, $\Delta h_{i}={\left[\Delta h_{1},\ldots ,\Delta h_{n}\right]}^{T}$.

The first Lyapunov function be constructed:

(23) $$V_{0}=\sum_{i=1}^{N}V_{i,0}=\sum_{i=1}^{N}\frac{1}{2}e_{i}^{T}P_{i}e_{i}.$$

By Lemma 1, we obtain:

(24) $$\begin{array}{cc} LV_{0} & \leq \sum_{i=1}^{N}\{\frac{1}{2}e_{i}^{T}\left(P_{i}A_{i}^{T}+A_{i}P_{i}\right)e_{i}+e_{i}^{T}P_{i}\left(\varepsilon_{i}+\Delta h_{i}\right)+\sum_{l=1}^{n}e_{i}^{T}P_{i}B_{i,l}\left[{\tilde{\psi }}_{i,l}^{T}\varphi_{i,l}\right]+B_{i,l}(\frac{1}{2}tr\left(F_{i}^{T}P_{i}F_{i}\right)\\ \; & +\frac{1}{2}\int_{R}\left(G_{i}^{T}P_{i}G_{i}+2e_{i}^{T}P_{i}G_{i}\right)\vartheta \left(d\zeta \right))\}\leq \sum_{i=1}^{N}\{-e_{i}^{T}Q_{i}e_{i}+e_{i}^{T}P_{i}\left(\varepsilon_{i}+\Delta h_{i}\right)\\ \; & +e_{i}^{T}P_{i}\sum_{l=1}^{n}B_{i,l}{\tilde{\psi }}_{i,l}^{T}\varphi_{i,l}+B_{i,l}\left(\frac{1}{2}tr\left(F_{i}^{T}P_{i}F_{i}\right)+\frac{1}{2}\int_{R}\left(G_{i}^{T}P_{i}G_{i}+2e_{i}^{T}P_{i}G_{i}\right)\vartheta \left(d\zeta \right)\right)\} \end{array}$$

Through Lemma 3 and Assumption 2, we obtain:

(25) $$\begin{array}{cc} \; & e_{i}^{T}P_{i}\left(\varepsilon_{i}+\Delta h_{i}\right)\\ \; & \leq \left|e_{i}^{T}P_{i}\varepsilon_{i}\right|+\left|e_{i}^{T}P_{i}\Delta g_{i}\right|\\ \; & \leq {∥e_{i}∥}^{2}+\frac{1}{2}{∥P_{i}\varepsilon_{i}∥}^{2}+\frac{1}{2}{∥P_{i}∥}^{2}\sum_{l=1}^{n}{\left|\Delta h_{i,l}\right|}^{2}\\ \; & \leq {∥e_{i}∥}^{2}+\frac{1}{2}{∥e_{i}∥}^{2}{∥P_{i}∥}^{2}\sum_{l=1}^{n}\gamma_{i,l}^{\;2}+\frac{1}{2}{∥P_{i}\varepsilon_{i}∥}^{2}\\ \; & \leq {∥e_{i}∥}^{2}\left(1+\frac{1}{2}{∥P_{i}∥}^{2}\sum_{l=1}^{n}\gamma_{i,l}^{2}\right)+\frac{1}{2}{∥P_{i}\varepsilon_{i}∥}^{2} \end{array}$$

and

(26) $$\begin{array}{cc} \; & e_{i}^{T}P_{i}\sum_{l=1}^{n}B_{i,l}{\tilde{\psi }}_{i,l}^{T}\varphi_{i,l}\left({\hat{X}}_{i,l}\right)\\ \; & \leq \frac{1}{2}e_{i}^{T}P_{i}^{T}P_{i}e_{i}+\frac{1}{2}\sum_{l=1}^{n}{\tilde{\psi }}_{i,l}^{T}\varphi_{i,l}\left({\hat{X}}_{i,l}\right)\varphi_{i,l}^{T}\left({\hat{X}}_{i,l}\right){\tilde{\psi }}_{i,l}\\ \; & \leq \frac{1}{2}\lambda_{i,\max}^{2}\left(P_{i}\right){∥e_{i}∥}^{2}+\frac{1}{2}\sum_{l=1}^{n}{\tilde{\psi }}_{i,l}^{T}{\tilde{\psi }}_{i,l} \end{array}$$

where $\lambda_{i,max}\left(P_{i}\right)$ is the maximum eigenvalue of the positive matrix $P_{i}$. According to (24)–(26), we can obtain that:

(27) $$\begin{array}{cc} LV_{0} & \leq \sum_{i=1}^{N}\{-q_{i,0}{∥e_{i}∥}^{2}+\frac{1}{2}{∥P_{i}\varepsilon_{i}^{q}∥}^{2}+\frac{1}{2}\sum_{l=1}^{n}{\tilde{\psi }}_{i,l}^{T}{\tilde{\psi }}_{i,l}\\ \; & +\frac{1}{2}\left(tr\left(F_{i}^{T}P_{i}F_{i}\right)+\int_{R}\left(G_{i}^{T}P_{i}G_{i}+2e_{i}^{T}P_{i}G_{i}\right)\vartheta \left(d\zeta \right)\right)\} \end{array}$$

**Assumption** **3** ([6,9])**.** *There are two known constants $\mu_{1}$, $\mu_{2}$, such that the stochastic noise parameters $F_{i}$, $G_{i}$ satisfy:*

(28) $$\begin{array}{cc} tr(F_{i}^{T}\left(X_{i,l},t\right)F_{i}\left(X_{i,l},t\right)) & \leq \mu_{1}{∥X_{i,l}∥}^{2}\\ \int_{R}G_{i}^{T}\left(X_{i,l},t,\zeta \right)G_{i}\left(X_{i,l},t,\zeta \right)\vartheta \left(d\zeta \right) & \leq \mu_{2}{∥X_{i,l}∥}^{2} \end{array}$$

According to Lemma 3 and Assumption 3, we can get that:

(29) $$\begin{array}{cc} \; & \frac{1}{2}tr\left(F_{i}^{T}P_{i}F_{i}\right)+\frac{1}{2}\int_{R}\left(G_{i}^{T}P_{i}G_{i}+2e_{i}^{T}P_{i}G_{i}\right)\vartheta \left(d\zeta \right)\\ \; & \leq \frac{\mu_{1}\lambda_{i,\max}^{2}\left(P_{i}\right)}{2}{∥X_{i,l}∥}^{2}+\mu_{2}\lambda_{i,\max}^{2}\left(P_{i}\right){∥X_{i,l}∥}^{2}\\ \; & +\frac{1}{2}\lambda_{i,\max}^{2}\left(P_{i}\right){∥e_{i}∥}^{2} \end{array}$$

so we have:

(30) $$\begin{array}{cc} LV_{0} & \leq \sum_{i=1}^{N}\{-q_{i,0}{∥e_{i}∥}^{2}+\frac{1}{2}{∥P_{i}\varepsilon_{i}∥}^{2}+\frac{1}{2}\sum_{l=1}^{n}{\tilde{\psi }}_{i,l}^{T}{\tilde{\psi }}_{i,l}\\ & +\mu \lambda_{i,\max}\left(P_{i}\right){∥X_{i,l}∥}^{2}\} \end{array}$$

where $q_{i,0}=\lambda_{i,\min}\left(P_{i}\right)-\left(1+\frac{1}{2}{∥P_{i}∥}^{2}\sum_{l=1}^{n}\gamma_{i,l}^{2}\right)$ and $\mu =\frac{\mu_{1}}{2}+\mu_{2}$.

Under Lemma 5, we can get that:

(31) $$\begin{array}{c} ∥X_{i,l}∥⩽\kappa \left(∥X_{i,l}\left(t_{0}\right)∥\right)e^{-℘\left(t-t_{0}\right)}+{℘}_{0} \end{array}$$

### 3.2. Controller Design

**Theorem** **1.** *With Assumptions 1–3, the system *(1)*, and the development of state observer *(16)*, virtual control laws *(37)–(39)*, together with adaptive laws *(41)–(43)*, compensation items *(33)–(36)* and an adaptive neural network controller through command-filtered method *(40)*, all signals $x_{i,1}$ in the MASs remain semi-global uniformly ultimately bounded (SGUUB) and the errors between outputs and the optimal solution are adequately small.*

**Proof.**  Define the error variables:

(32) $$\begin{array}{cc} \; & s_{i,1}=2m_{i}\left(x_{i,1}-b_{i}\right)+\sum_{j\in N_{i}}a_{ij}\left(x_{i,1}-x_{j,1}\right)\\ & s_{i,l}={\hat{x}}_{i,l}-{\bar{a}}_{i,l}\\ & z_{i,l}=s_{i,l}-\xi_{i,l} \end{array}$$

where $b_{i}=-1/2m_{i}\tau_{i}$, $s_{i,l}$ represents the tracking error, ${\bar{a}}_{i,l}$ is the command filter output which is relative to the virtual controller $a_{i,l}$, and $\xi_{i,l}$ is the error compensation signal, designed as:

(33) $$\begin{array}{c} {\dot{\xi }}_{i,1}=d_{i}\left(\xi_{i,2}+{\bar{a}}_{i,2}-a_{i,1}\right)-c_{i,1}\xi_{i,1}-\xi_{i,1} \end{array}$$

(34) $${\dot{\xi }}_{i,2}={\bar{a}}_{i,3}-a_{i,2}-d_{i}\xi_{i,1}+\xi_{i,3}-c_{i,2}\xi_{i,2}-\frac{3}{2}\xi_{i,2}.$$

(35) $${\dot{\xi }}_{i,m}={\bar{a}}_{i,m}-a_{i,m}-\xi_{i,m-1}+\xi_{i,m+1}-c_{i,m}\xi_{i,m}-\frac{3}{2}\xi_{i,m}$$

(36) $${\dot{\xi }}_{i,n}=-\xi_{i,n-1}-c_{i,n}\xi_{i,n}-\frac{3}{2}\xi_{i,n}$$

The structure of the virtual controllers and the control input are as follows:

(37) $$\begin{array}{cc} a_{i,1} & =\frac{1}{d_{i}}(2m_{i}{\dot{b}}_{i}-c_{i,1}s_{i,1}-s_{i,1}\\ \; & +\sum_{j\in N_{i}}a_{ij}\left({\hat{x}}_{j,2}+\theta_{j,1}^{T}\varphi_{j,1}\right))-\theta_{i,1}^{T}\varphi_{i,1} \end{array}$$

(38) $$a_{i,2}={\dot{\bar{a}}}_{i,2}-d_{i}s_{i,1}-c_{i,2}s_{i,2}-\frac{3}{2}s_{i,2}-\psi_{i,2}^{T}\varphi_{i,2}\left({\hat{X}}_{i,2}\right)$$

(39) $$a_{i,m}={\dot{\bar{a}}}_{i,m}-s_{i,m-1}-c_{i,m}s_{i,m}-\frac{3}{2}s_{i,m}-\psi_{i,m}^{T}\varphi_{i,m}\left({\hat{X}}_{i,m}\right)$$

(40) $$u_{i}={\dot{\bar{a}}}_{i,n}-s_{i,n-1}-c_{i,n}s_{i,n}-\frac{3}{2}s_{i,n}-\psi_{i,n}^{T}\varphi_{i,n}\left({\hat{X}}_{i,n}\right)$$

where $d_{i}=2m_{i}+\sum_{j\in N_{i}}a_{ij}$, and $c_{i,l},1\leq l\leq n$ are the parameters that need to be designed.

Design the adaptive laws as:

(41) $$\begin{array}{c} {\dot{\psi }}_{i,1}=r_{i,1}d_{i}\varphi_{i,1}z_{i,1}-{\bar{r}}_{i,1}\psi_{i,1} \end{array}$$

(42) $$\begin{array}{c} {\dot{\psi }}_{j,1}=-r_{j,1}\varphi_{j,1}z_{j,1}-{\bar{r}}_{j,1}\psi_{j,1} \end{array}$$

(43) $${\dot{\psi }}_{i,l}=r_{i,l}\varphi_{i,l}z_{i,l}-{\bar{r}}_{i,l}\psi_{i,l}.$$

where 2≤l≤n, $r_{i,1}$, ${\bar{r}}_{i,1}$, $r_{j,1}$, ${\bar{r}}_{j,1}$, $r_{i,l}$ and ${\bar{r}}_{i,l}$ are positive design constants.

□

#### 3.2.1. Step 1

Firstly, according to (7), the gradient of the penalty function can be calculated:

(44) $$\frac{\partial P(x_{1})}{\partial x_{1}}=vec\left(\frac{\partial {£}_{i}\left(x_{i,1}\left(t\right)\right)}{\partial x_{i,1}}\right)+Lx_{1}$$

where $vec\left(\frac{\partial {£}_{i}\left(x_{i,1}\left(t\right)\right)}{\partial x_{i,1}}\right)$ is a column vector. The optimal solution $x_{1}^{*}$ satisfies:

$$\begin{array}{c} \frac{\partial P(x_{1}^{*})}{\partial x_{1}^{*}}=0. \end{array}$$

So, for agent *i*:

(45) $$\frac{\partial {£}_{i}\left(x_{i,1}^{*}\left(t\right)\right)}{\partial x_{i,1}^{*}}+\sum_{j\in N_{i}}a_{ij}\left(x_{i,1}^{*}-x_{j,1}^{*}\right)=0.$$

Under (9) and (45),we will obtain that:

(46) $$2m_{i}\left(x_{i,1}^{*}-b_{i}\right)+\sum_{j\in N_{i}}a_{ij}\left(x_{i,1}^{*}-x_{j,1}^{*}\right)=0.$$

Then, combine the (32) with (46), we can get:

(47) $$\begin{array}{cc} \frac{\partial P(x_{1})}{\partial x_{i,1}} & =\frac{\partial {£}_{i}\left(x_{i,1}\left(t\right)\right)}{\partial x_{i,1}}+\sum_{j\in N_{i}}a_{ij}\left(x_{i,1}-x_{j,1}\right)\\ & =2m_{i}\left(x_{i,1}-b_{i}\right)+\sum_{j\in N_{i}}a_{ij}\left(x_{i,1}-x_{j,1}\right)\\ & =s_{i,1} \end{array}$$

The Lyapunov function be constructed as:

(48) $$\begin{array}{cc} \; & V_{1}=V_{0}+\sum_{i=1}^{N}\left(\frac{1}{2}z_{i,1}^{2}+\frac{1}{2r_{i,1}}{\tilde{\psi }}_{i,1}^{T}{\tilde{\psi }}_{i,1}+\frac{1}{2}\sum_{j\in N_{i}}a_{ij}\frac{1}{r_{j,1}}{\tilde{\psi }}_{j,1}^{T}{\tilde{\psi }}_{j,1}\right) \end{array}$$

where $z_{1}={\left[z_{1,1}\;\cdots \;z_{N,1}\right]}^{T}$, $r_{i,1}$ and $r_{j,1}$ are designed parameters. According to (1), (16) and (32), we have:

(49) $$\begin{array}{cc} ds_{i,1}= & (\left(2m_{i}+\sum_{j\in N_{i}}a_{ij}\right)dx_{i,1}-\sum_{j\in N_{i}}a_{ij}\left(x_{j,2}+h_{j,1}\left(x_{j,1}\right)\right))dt\\ \; & +\left(2m_{i}+\sum_{j\in N_{i}}a_{ij}\right)\left(F_{i,1}dw\left(t\right)+\int_{R}G_{i,1}N\left(dt,d\zeta \right)\right)\\ \; & -\sum_{j\in N_{i}}a_{ij}\left(F_{j,1}dw\left(t\right)+\int_{R}G_{j,1}N\left(dt,d\zeta \right)\right)-2m_{i}b_{i}dt\\ = & (d_{i}\left(z_{i,2}+\xi_{i,2}+e_{i,2}+{\bar{a}}_{i,2}\right)-2m_{i}b_{i}dt+d_{i}h_{i,1}\left(x_{i,1}\right)\\ \; & -\sum_{j\in N_{i}}a_{ij}\left({\hat{x}}_{j,2}+e_{j,2}\right)-\sum_{j\in N_{i}}a_{ij}h_{j,1}\left(x_{j,1}\right))dt\\ \; & +d_{i}\left(F_{i,1}dw\left(t\right)+\int_{R}G_{i,1}N\left(dt,d\zeta \right)\right)\\ \; & -\sum_{j\in N_{i}}a_{ij}\left(F_{j,1}dw\left(t\right)+\int_{R}G_{j,1}N\left(dt,d\zeta \right)\right) \end{array}$$

where $d_{i}=2m_{i}+\sum_{j\in N_{i}}a_{ij}$. By (48), we can get ${\dot{z}}_{i,1}={\dot{s}}_{i,1}-{\dot{\xi }}_{i,1}$. Then, according to (48), (49) and Lemma 1, we can obtain:

(50) $$\begin{array}{cc} LV_{1}= & LV_{0}+\sum_{i=1}^{N}\{z_{i,1}(d_{i}\left(z_{i,2}+\xi_{i,2}+e_{i,2}+{\bar{a}}_{i,2}\right)-2m_{i}b_{i}dt-{\dot{\xi }}_{i,1}+d_{i}h_{i,1}\left(x_{i,1}\right)\\ \; & -\sum_{j\in N_{i}}a_{ij}\left({\hat{x}}_{j,2}+e_{j,2}+h_{j,1}\left(x_{j,1}\right)\right))-\frac{1}{r_{i,1}}{\tilde{\psi }}_{i,1}^{T}{\dot{\psi }}_{i,1}-\sum_{j\in N_{i}}a_{ij}\frac{1}{r_{j,1}}{\tilde{\psi }}_{j,1}^{T}{\dot{\psi }}_{j,1}+\frac{d_{i}}{2}(tr\left(F_{i,1}^{T}F_{i,1}\right)\\ \; & +\int_{R}G_{i,1}^{T}G_{i,1}\vartheta \left(d\zeta \right))-\frac{\sum_{j\in N_{i}}a_{ij}}{2}\left(tr\left(F_{j,1}^{T}F_{j,1}\right)+\int_{R}G_{j,1}^{T}G_{j,1}\vartheta \left(d\zeta \right)\right)\} \end{array}$$

Applying Lemma 3, the following inequality holds:

(51) $$\begin{array}{cc} \; & z_{i,1}d_{i}e_{i,2}+z_{i,1}\sum_{j\in N_{i}}\left(-a_{ij}e_{j,2}\right)\\ \; & \leq z_{i,1}^{2}+\frac{d_{i}^{2}}{2}{∥e_{i,2}∥}^{2}+\frac{{\left(\sum_{j\in N_{i}}a_{ij}\right)}^{2}}{2}{∥e_{j,2}∥}^{2} \end{array}$$

From Assumption 3, there are four of known constants $\eta_{1}$, $\eta_{2}$, $\eta_{3}$, $\eta_{4}$ such that the parameters of stochastic noises $F_{i,1}$, $G_{i,1}$, $F_{j,1}$, $G_{j,1}$ satisfy:

(52) $$\begin{array}{cc} tr\left(F_{i,1}^{T}\left(X_{i,l},t\right)F_{i,1}\left(X_{i,l},t\right)\right) & ⩽\eta_{1}{∥X_{i,l}∥}^{2}\\ \int_{R}G_{i,1}^{T}\left(X_{i,l},t,\zeta \right)G_{i,1}\left(X_{i,l},t,\zeta \right)\vartheta \left(d\zeta \right) & ⩽\eta_{2}{∥X_{i,l}∥}^{2}\\ tr\left(F_{j,1}^{T}\left(X_{j,l},t\right)F_{j,1}\left(X_{j,l},t\right)\right) & ⩽\eta_{3}{∥X_{j,l}∥}^{2}\\ \int_{R}G_{j,1}^{T}\left(X_{j,l},t,\zeta \right)G_{j,1}\left(X_{j,l},t,\zeta \right)\vartheta \left(d\zeta \right) & ⩽\eta_{4}{∥X_{j,l}∥}^{2} \end{array}$$

Based on the first virtual controller $a_{i,1}$ (37), the error compensation signal $\xi_{i,1}$ (33) and update law $\psi_{i,1}$ (41), $\psi_{j,1}$ (42), we will get that:

(53) $$\begin{array}{cc} LV_{1} & \leq -q_{1}\left||e\right|{|}^{2}+\sum_{i=1}^{N}\{\frac{1}{2}\left|\right|P_{i}\varepsilon_{i}|{|}^{2}+\frac{1}{2}\sum_{l=1}^{n}{\tilde{\psi }}_{i,l}^{T}{\tilde{\psi }}_{i,l}+\mu \lambda_{i,\max}\left(P_{i}\right)\left|\right|X_{i,l}|{|}^{2}\\ \; & +d_{i}z_{i,1}z_{i,2}-c_{i,1}z_{i,1}^{2}+\frac{{\bar{r}}_{i,1}}{r_{i,1}}{\tilde{\psi }}_{i,1}^{T}\psi_{i,1}+\sum_{j\in N_{I}}a_{ij}\frac{{\bar{r}}_{j,1}}{r_{j,1}}{\tilde{\psi }}_{j,1}^{T}\psi_{j,1}+D_{i,1}\} \end{array}$$

From Young’s inequality, we will get:

(54) $$\begin{array}{cc} {\tilde{\psi }}_{i,1}^{T}\psi_{i,1} & \leq -\frac{1}{2}{\tilde{\psi }}_{i,1}^{T}{\tilde{\psi }}_{i,1}+\frac{1}{2}\psi_{i,1}^{*T}\psi_{i,1}^{*}\\ {\tilde{\psi }}_{j,1}^{T}\psi_{j,1} & \leq -\frac{1}{2}{\tilde{\psi }}_{j,1}^{T}{\tilde{\psi }}_{j,1}+\frac{1}{2}\psi_{j,1}^{*T}\psi_{j,1}^{*} \end{array}$$

Therefore, rewrite (53) as:

(55) $$\begin{array}{cc} LV_{1} & \leq -q_{1}\left||e\right|{|}^{2}+\sum_{i=1}^{N}\{\frac{1}{2}\left|\right|P_{i}\varepsilon_{i}|{|}^{2}+\frac{1}{2}\sum_{l=1}^{n}{\tilde{\psi }}_{i,l}^{T}{\tilde{\psi }}_{i,l}+\mu \lambda_{i,\max}\left(P_{i}\right)\left|\right|X_{i,l}|{|}^{2}\\ \; & +d_{i}z_{i,1}z_{i,2}-c_{i,1}z_{i,1}^{2}-\frac{{\bar{r}}_{i,1}}{2r_{i,1}}{\tilde{\psi }}_{i,1}^{T}{\tilde{\psi }}_{i,1}-\sum_{j\in N_{I}}a_{ij}\frac{{\bar{r}}_{j,1}}{2r_{j,1}}{\tilde{\psi }}_{j,1}^{T}{\tilde{\psi }}_{j,1}+D_{i,1}\} \end{array}$$

where $e={\left[e_{1}^{T},\cdots ,e_{n}^{T}\right]}^{T}$, $q_{1}=\sum_{i=1}^{N}\left(q_{i,0}-\frac{1}{2}d_{i}^{2}-\frac{{\left(\sum_{j\in N_{i}}a_{ij}\right)}^{2}}{2}\right)$ and $D_{i,1}=\frac{d_{i}}{2}\left(\eta_{1}+\eta_{2}\right){∥X_{i,l}∥}^{2}+\frac{\sum_{j\in N_{i}}a_{ij}}{2}\left(\eta_{3}+\eta_{4}\right){∥X_{j,l}∥}^{2}+\frac{{\bar{r}}_{i,1}}{2r_{i,1}}\psi_{i,1}^{*T}\psi_{i,1}^{*}+\sum_{j\in N_{I}}a_{ij}\frac{{\bar{r}}_{j,1}}{2r_{j,1}}\psi_{j,1}^{*T}\psi_{j,1}^{*}$.

#### 3.2.2. Step 2

In accordance with (32), we take $z_{i,2}=s_{i,2}-\xi_{i,2}$. After (16) and (18), we have:

(56) $$\begin{array}{cc} {\dot{z}}_{i,2} & ={\dot{s}}_{i,2}-{\dot{\xi }}_{i,2}\\ \; & =z_{i,3}+\xi_{i,3}+{\bar{a}}_{i,3}-{\dot{\bar{a}}}_{i,2}-{\dot{\xi }}_{i,2}+\iota_{i,2}e_{i,1}\\ \; & +\psi_{i,2}^{T}\varphi_{i,2}+{\tilde{\psi }}_{i,2}^{T}\varphi_{i,2}+\varepsilon_{i,2}+\Delta h_{i,2} \end{array}$$

Construct the Lyapunov function:

(57) $$\begin{array}{c} V_{2}=V_{1}+\sum_{i=1}^{N}\left(\frac{1}{2}z_{i,2}^{2}+\frac{1}{2r_{i,2}}{\tilde{\psi }}_{i,2}^{T}{\tilde{\psi }}_{i,2}\right) \end{array}$$

Then, we have:

(58) $$\begin{array}{c} LV_{2}=LV_{1}+\sum_{i=1}^{N}\left(z_{i,2}{\dot{z}}_{i,2}+\frac{1}{r_{i,2}}{\tilde{\psi }}_{i,2}^{T}{\dot{\tilde{\psi }}}_{i,2}\right) \end{array}$$

where $r_{i,2}$ is a positive designed parameter. Based (56) and (58), we can obtain that:

(59) $$\begin{array}{cc} LV_{2} & =LV_{1}+\sum_{i=1}^{N}\left\{z_{i,2}{\dot{z}}_{i,2}+\frac{1}{r_{i,2}}{\tilde{\psi }}_{i,2}^{T}{\dot{\tilde{\psi }}}_{i,2}\right\}\\ \; & =LV_{1}+\sum_{i=1}^{N}\{z_{i,2}(z_{i,3}+\xi_{i,3}+{\bar{a}}_{i,3}-{\dot{\bar{a}}}_{i.2}\\ \; & -{\dot{\xi }}_{i,2}+\iota_{i,2}e_{i,1}+\psi_{i,2}^{T}\varphi_{i,2}+{\tilde{\psi }}_{i,2}^{T}\varphi_{i,2}+\varepsilon_{i,2}\\ \; & +\Delta h_{i,2})-\frac{1}{r_{i,2}}{\tilde{\psi }}_{i,2}^{T}{\dot{\psi }}_{i,2}\} \end{array}$$

According to Lemma 3, we obtain:

(60) $$\iota_{i,2}z_{i,2}e_{i,1}\leq \frac{1}{2}z_{i,2}^{2}+\frac{1}{2}\iota_{i,2}^{2}{∥e_{i,1}∥}^{2}$$

(61) $$z_{i,2}\left(\varepsilon_{i,2}+\Delta h_{i,2}\right)\leq z_{i,2}^{2}+\frac{1}{2}{∥\varepsilon_{i,2}∥}^{2}+\frac{1}{2}\gamma_{i,2}^{2}{∥e_{i,2}∥}^{2}$$

Substituting the second virtual controller $a_{i,2}$ (38), the error compensation signal $\xi_{i,2}$ (34) and update laws $\psi_{i,2}$ (43) and the inequalities (60), (61) into (59), we will calculate that:

(62) $$\begin{array}{cc} LV_{2} & \leq LV_{1}+\sum_{i=1}^{N}\{z_{i,2}z_{i,3}-c_{i,2}z_{i.2}^{2}-d_{i}z_{i,1}z_{i,2}\\ \; & +\frac{1}{2}\left(\iota_{i,2}^{2}{∥e_{i,1}∥}^{2}+{∥\varepsilon_{i,2}∥}^{2}+\gamma_{i,2}^{2}{∥e_{i,2}∥}^{2}\right)+\frac{{\bar{r}}_{i,2}}{r_{i,2}}{\tilde{\psi }}_{i,2}^{T}\psi_{i,2}\}\\ \; & \leq -q_{2}{∥e∥}^{2}+\sum_{i=1}^{N}\{\frac{1}{2}{∥P_{i}\varepsilon_{i}∥}^{2}+\frac{1}{2}\sum_{l=1}^{n}{\tilde{\psi }}_{i,l}^{T}{\tilde{\psi }}_{i,l}+\mu \lambda_{i,\max}\left(P_{i}\right){∥X_{i,l}∥}^{2}\\ \; & +z_{i,2}z_{i,3}-\sum_{l=1}^{2}c_{i,l}z_{i.l}^{2}+\sum_{l=1}^{2}\frac{{\bar{r}}_{i,l}}{r_{i,l}}{\tilde{\psi }}_{i,l}^{T}\psi_{i,l}+D_{i,2}\} \end{array}$$

From Young’s inequality, we will get:

(63) $$\begin{array}{c} {\tilde{\psi }}_{i,l}^{T}\psi_{i,l}\leq -\frac{1}{2}{\tilde{\psi }}_{i,l}^{T}{\tilde{\psi }}_{i,l}+\frac{1}{2}\psi_{i,l}^{*T}\psi_{i,l}^{*} \end{array}$$

Therefore, rewrite (62) as:

(64) $$\begin{array}{cc} LV_{2} & \leq -q_{2}{∥e∥}^{2}+\sum_{i=1}^{N}\{\frac{1}{2}{∥P_{i}\varepsilon_{i}∥}^{2}+\frac{1}{2}\sum_{l=1}^{n}{\tilde{\psi }}_{i,l}^{T}{\tilde{\psi }}_{i,l}+\mu \lambda_{i,\max}\left(P_{i}\right){∥X_{i,l}∥}^{2}\\ \; & +z_{i,2}z_{i,3}-\sum_{l=1}^{2}c_{i,l}z_{i.l}^{2}-\sum_{l=1}^{2}\frac{{\bar{r}}_{i,l}}{2r_{i,l}}{\tilde{\psi }}_{i,l}^{T}{\tilde{\psi }}_{i,l}+D_{i,2}\} \end{array}$$

where $q_{2}=q_{1}-\sum_{i=1}^{N}\frac{1}{2}\left(\iota_{i,2}^{2}+\gamma_{i,2}^{2}\right)$, $D_{i,2}=D_{i,1}+\frac{1}{2}{∥\varepsilon_{i,2}∥}^{2}+\frac{{\bar{r}}_{i,2}}{2r_{i,2}}\psi_{i,2}^{*T}\psi_{i,2}^{*}$.

#### 3.2.3. Step m

According to (32), we can get:

(65) $$\begin{array}{cc} {\dot{z}}_{i,m} & =z_{i,m+1}+\xi_{i,m+1}+{\bar{a}}_{i,m+1}-{\dot{\bar{a}}}_{i,m}-{\dot{\xi }}_{i,m}+\iota_{i,m}e_{i,1}\\ \; & +\psi_{i,m}^{T}\varphi_{i,m}+{\tilde{\psi }}_{i,m}^{T}\varphi_{i,m}+\varepsilon_{i,m}+\Delta h_{i,m} \end{array}$$

Put forward the Lyapunov function:

(66) $$\begin{array}{c} V_{m}=V_{m-1}+\sum_{i=1}^{N}\left(\frac{1}{2}z_{i,m}^{2}+\frac{1}{2r_{i,m}}{\tilde{\psi }}_{i,m}^{T}{\tilde{\psi }}_{i,m}\right) \end{array}$$

where $r_{i,m}$ is a positive designed parameter. After derivation:

(67) $$\begin{array}{c} LV_{m}=LV_{m-1}+\sum_{i=1}^{N}\left(z_{i,m}{\dot{z}}_{i,m}+\frac{1}{r_{i,m}}{\tilde{\psi }}_{i,m}^{T}{\dot{\tilde{\psi }}}_{i,m}\right) \end{array}$$

Substituting (66) into (67), we can get:

(68) $$\begin{array}{cc} LV_{m} & =LV_{m-1}+\sum_{i=1}^{N}\{z_{i,m}(z_{i,m+1}+\xi_{i,m+1}+{\bar{a}}_{i,m+1}\\ \; & -{\dot{\bar{a}}}_{i,m}-{\dot{\xi }}_{i,m}+\iota_{i,m}e_{i,1}+\psi_{i,m}^{T}\varphi_{i,m}+{\tilde{\psi }}_{i,m}^{T}\varphi_{i,m}\\ \; & +\varepsilon_{i,m}+\Delta h_{i,m})+\frac{1}{r_{i,m}}{\tilde{\psi }}_{i,m}^{T}{\dot{\tilde{\psi }}}_{i,m}\} \end{array}$$

According to Lemma 3, we obtain:

(69) $$\iota_{i,m}e_{i,1}z_{i,m}\leq \frac{1}{2}z_{i,m}^{2}+\frac{1}{2}\iota_{i,m}^{2}{∥e_{i,1}∥}^{2}$$

(70) $$z_{i,m}\left(\varepsilon_{i,m}+\Delta h_{i,m}\right)\leq z_{i,m}^{2}+\frac{1}{2}{∥\varepsilon_{i,m}∥}^{2}+\frac{1}{2}\gamma_{i,m}^{2}{∥e_{i,m}∥}^{2}$$

On the basis of the m-th virtual controller $a_{i,m}$ (39), the error compensation signal $\xi_{i,m}$ (35) the update laws $\psi_{i,m}$ (43) the above inequalities, we will obtain that:

(71) $$\begin{array}{cc} LV_{m} & \leq -q_{m}{∥e∥}^{2}+\sum_{i=1}^{N}\{\frac{1}{2}{∥P_{i}\varepsilon_{i}∥}^{2}+\frac{1}{2}\sum_{l=1}^{n}{\tilde{\psi }}_{i,l}^{T}{\tilde{\psi }}_{i,l}+\mu \lambda_{i,\max}\left(P_{i}\right){∥X_{i,l}∥}^{2}\\ \; & +z_{i,m}z_{i,m+1}-\sum_{l=1}^{m}c_{i,l}z_{i,l}^{2}+\sum_{l=1}^{m}\frac{{\bar{r}}_{i,l}}{r_{i,l}}{\tilde{\psi }}_{i,l}^{T}\psi_{i,l}+D_{i,m}\} \end{array}$$

From Young’s inequality, we will get:

(72) $$\begin{array}{c} {\tilde{\psi }}_{i,l}^{T}\psi_{i,l}\leq -\frac{1}{2}{\tilde{\psi }}_{i,l}^{T}{\tilde{\psi }}_{i,l}+\frac{1}{2}\psi_{i,l}^{*T}\psi_{i,l}^{*} \end{array}$$

Therefore, rewrite (71) as:

(73) $$\begin{array}{cc} LV_{m} & \leq -q_{m}{∥e∥}^{2}+\sum_{i=1}^{N}\{\frac{1}{2}{∥P_{i}\varepsilon_{i}∥}^{2}+\frac{1}{2}\sum_{l=1}^{n}{\tilde{\psi }}_{i,l}^{T}{\tilde{\psi }}_{i,l}+\mu \lambda_{i,\max}\left(P_{i}\right){∥X_{i,l}∥}^{2}\\ \; & +z_{i,m}z_{i,m+1}-\sum_{l=1}^{m}c_{i,l}z_{i,l}^{2}-\sum_{l=1}^{m}\frac{{\bar{r}}_{i,l}}{2r_{i,l}}{\tilde{\psi }}_{i,l}^{T}{\tilde{\psi }}_{i,l}+D_{i,m}\} \end{array}$$

where $q_{m}=q_{m-1}-\sum_{i=1}^{N}\frac{1}{2}\left(\iota_{i,m}^{2}+\gamma_{i,m}^{2}\right)$, $D_{i,m}=D_{i,m-1}+\frac{1}{2}{∥\varepsilon_{i,m}∥}^{2}+\frac{{\bar{r}}_{i,m}}{2r_{i,m}}\psi_{i,m}^{*T}\psi_{i,m}^{*}$.

#### 3.2.4. Step n

According to (32), we can get:

(74) $$\begin{array}{cc} {\dot{z}}_{i,n} & =u_{i}+\iota_{i,n}e_{i,1}+\psi_{i,n}^{T}\varphi_{i,n}+{\tilde{\psi }}_{i,n}^{T}\varphi_{i,n}\\ \; & +\varepsilon_{i,n}+\Delta h_{i,n}-{\dot{\bar{a}}}_{i.n}-{\dot{\xi }}_{i,n} \end{array}$$

The Lyapunov function can be constructed as:

(75) $$\begin{array}{c} V_{n}=V_{n-1}+\sum_{i=1}^{N}\left\{\frac{1}{2}z_{i,n}^{2}+\frac{1}{2r_{i,n}}{\tilde{\psi }}_{i,n}^{T}{\tilde{\psi }}_{i,n}\right\} \end{array}$$

where $r_{i,n}$ is a positive designed parameter. Under (74) and (75), we obtain:

(76) $$\begin{array}{cc} LV_{n} & =LV_{n-1}+\sum_{i=1}^{N}\left\{z_{i,n}{\dot{z}}_{i,n}+\frac{1}{r_{i,n}}{\tilde{\psi }}_{i,n}^{T}{\dot{\tilde{\psi }}}_{i,n}\right\}\\ \; & =LV_{n-1}+\sum_{i=1}^{N}\{z_{i,n}(u_{i}+\iota_{i,n}e_{i,1}+\psi_{i,n}^{T}\psi_{i,n}\\ \; & +{\tilde{\psi }}_{i,n}^{T}\psi_{i,n}+\varepsilon_{i,n}+\Delta h_{i,n}-{\dot{\bar{a}}}_{i.n}-{\dot{\xi }}_{i,n})\\ \; & +\frac{1}{r_{i,n}}{\tilde{\psi }}_{i,n}^{T}{\dot{\tilde{\psi }}}_{i,n}\} \end{array}$$

Under Lemma 3, we will taken the inequality relation as follows:

(77) $$\iota_{i,n}e_{i,1}z_{i,n}\leq \frac{1}{2}z_{i,n}^{2}+\frac{1}{2}\iota_{i,n}^{2}{∥e_{i,1}∥}^{2}$$

(78) $$z_{i,n}\left(\varepsilon_{i,n}+\Delta h_{i,n}\right)\leq z_{i,n}^{2}+\frac{1}{2}{∥\varepsilon_{i,n}∥}^{2}+\frac{1}{2}\gamma_{i,n}^{2}{∥e_{i,n}∥}^{2}$$

Under (77) and (78), substituting the *n*-th control controller $u_{i}$ (40), the error compensation signal $\xi_{i,n}$ (36), and the update laws $\psi_{i,n}$ (43) into (76), we will get that:

(79) $$\begin{array}{cc} LV_{n} & \leq -q_{n}{∥e∥}^{2}+\sum_{i=1}^{N}\{\frac{1}{2}{∥P_{i}\varepsilon_{i}∥}^{2}+\frac{1}{2}\sum_{l=1}^{n}{\tilde{\psi }}_{i,l}^{T}{\tilde{\psi }}_{i,l}+\mu \lambda_{i,\max}\left(P_{i}\right){∥X_{i,l}∥}^{2}\\ \; & -\sum_{l=1}^{n}c_{i,l}z_{i,l}^{2}+\sum_{l=1}^{n}\frac{{\bar{r}}_{i,l}}{r_{i,l}}{\tilde{\psi }}_{i,l}^{T}\psi_{i,l}+D_{i,n}\} \end{array}$$

From Young’s inequality, we will get:

(80) $$\begin{array}{c} {\tilde{\psi }}_{i,l}^{T}\psi_{i,l}\leq -\frac{1}{2}{\tilde{\psi }}_{i,l}^{T}{\tilde{\psi }}_{i,l}+\frac{1}{2}\psi_{i,l}^{*T}\psi_{i,l}^{*} \end{array}$$

Therefore, rewrite (77) as:

(81) $$\begin{array}{cc} LV_{n} & \leq -q_{n}{∥e∥}^{2}+\sum_{i=1}^{N}\{-\sum_{l=1}^{n}c_{i,l}z_{i,l}^{2}+\frac{1}{2}\sum_{l=1}^{n}{\tilde{\psi }}_{i,l}^{T}{\tilde{\psi }}_{i,l}-\sum_{l=1}^{n}\frac{{\bar{r}}_{i,l}}{2r_{i,l}}{\tilde{\psi }}_{i,l}^{T}{\tilde{\psi }}_{i,l}\\ \; & +\mu \lambda_{i,\max}\left(P_{i}\right){∥X_{i,l}∥}^{2}+\frac{1}{2}{∥P_{i}\varepsilon_{i}∥}^{2}+D_{i,n}\} \end{array}$$

where $q_{n}=q_{n-1}-\sum_{i=1}^{N}\frac{1}{2}\left(\iota_{i,n}^{2}+\gamma_{i,n}^{2}\right)$, $D_{i,n}=D_{i,n-1}+\frac{1}{2}{∥\varepsilon_{i,n}∥}^{2}+\frac{{\bar{r}}_{i,n}}{2r_{i,n}}\psi_{i,n}^{*T}\psi_{i,n}^{*}$.

### 3.3. Stability Analysis

From (81), we will note that $I=D_{i,n-1}+\frac{1}{2}{∥\varepsilon_{i,n}∥}^{2}+\mu \lambda_{i,\max}\left(P_{i}\right){∥X_{i,l}∥}^{2}+\frac{{\bar{r}}_{i,m}}{2r_{i,m}}\psi_{i,m}^{*T}\psi_{i,m}^{*}+\frac{1}{2}{∥P_{i}\varepsilon_{i}∥}^{2}$, and according to Lemma 5, we will obtain $∥X_{i,l}∥⩽\kappa \left(∥X_{i,l}\left(t_{0}\right)∥\right)e^{-℘\left(t-t_{0}\right)}+{℘}_{0}$.

Define $ℑ=\min\{2q_{n}/\lambda_{i,\max}\left(P_{i}\right),2\sum_{l=1}^{n}c_{i,l},\sum_{l=1}^{n}\left(\frac{{\bar{r}}_{i,l}}{r_{i,l}}+\frac{1}{2}\right)\}$, Equation (81) then becomes:

(82) $$LV\left(x,t\right)\leq -ℑV\left(x,t\right)+I.$$

Therefore, we can further write (82) as:

(83) $$d\left(E\left[V\left(x,t\right)\right]\right)/dt=E\left[L\left[V\right]\right]⩽-ℑE\left[V\right]+I$$

where $E\left[•\right]$ is the probability expectation. Let ℑ>I/K, $E\left[V\right]=K$; later, we will obtain that $d\left(E\left[V\right]\right)/dt<0$. Accordingly, V⩽K which is an invariant set, which means that if $E\left[V\left(x\left(t_{0}\right),t_{0}\right)\right]⩽K$, later $E\left[V\left(x,t\right)\right]⩽K$ for all time $t\in \left[t_{0},t_{\rho }\right]$. Hence, (83) with regards to any $V\left(x\left(t_{0}\right),t_{0}\right)<K$, and all time $t\in \left[t_{0},t_{\rho }\right]$. Moreover, it holds that:

(84) $$E\left[V\left(x\left(t_{\rho }\right),t_{\rho }\right)\right]⩽EV\left(x\left(t_{0}\right),t_{0}\right)+I\left(t_{\rho }-t_{0}\right)⩽\overset{⌣}{c}e^{t},$$

where $\overset{⌣}{c}=\left(EV\left(x\left(t_{0}\right),t_{0}\right)+I\right)/e^{t_{0}}$, then, we will get that:

(85) $$\begin{array}{cc} EV\left(x(t),t\right) & \leq e^{-ℑ\left(t-t_{0}\right)}EV\left(x\left(t_{0}\right),t_{0}\right)+I/ℑ-I/ℑe^{-ℑ\left(t-t_{0}\right)}\\ \; & \leq EV\left(x\left(t_{0}\right),t_{0}\right)+I/ℑ \end{array}$$

According to Lemma 4, we can rewrite (85) as:

(86) $$0⩽E\left[LV\left(x,t\right)\right]⩽e^{-ℑt}V\left(x\left(t_{0}\right),t_{0}\right)+I/ℑ.$$

Based on (86), it can be inferred that $E\left[V\left(x,t\right)\right]$ is ultimately bounded by I/ℑ, we obtain:

(87) $$\lim_{t\to \infty }E\left[V\left(x,t\right)\right]⩽I/ℑ.$$

After that, we can get that all the variables, such as $x_{i,n}$, *e*, $s_{i,l}$, $z_{i,l}$, the virtual control $a_{i,l}$, and the control inputs $u_{i}$ are bounded in probability on the basis of the Lyapunov function. Likewise, we can sum up that all signals of MASs (1) remain SGUUB in the closed-loop system and the errors between outputs and the optimal value is sufficiently small.

**Remark** **2.** *Compared to [45], in which the DOP is investigated for MASs with the nonlinear function, the high-order MASs in this paper contain stochastic noise, which implies that the control protocol designed will be incorporated into many commercial engineering applications, such as marine surface vehicles, unmanned aerial vehicles, and wheeled multi-mobile robots.*

## 4. Simulation

**Figure 1:**
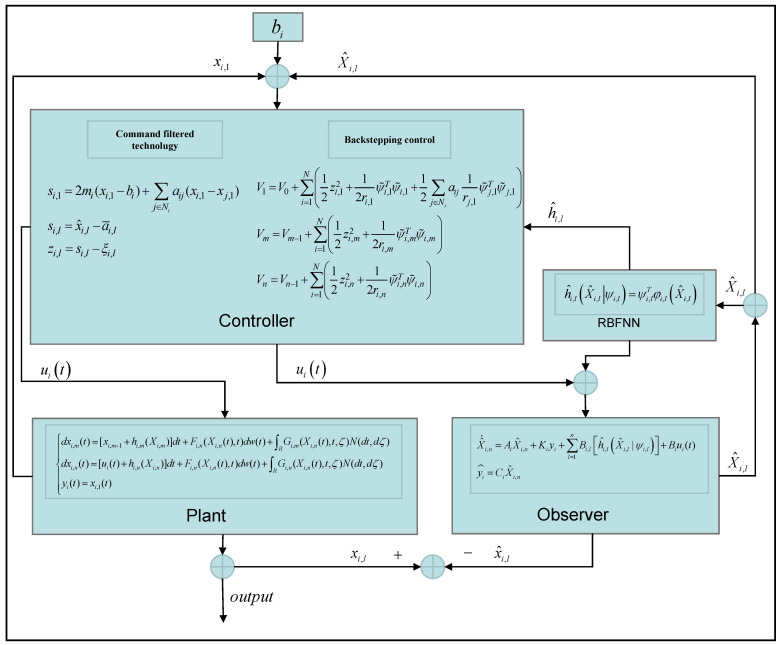
The block diagram of the designed control system.

To illustrate the proposed method, simulations are performed in this section. Figure 1 shows the block diagram of the designed control system.

**Figure 2:**
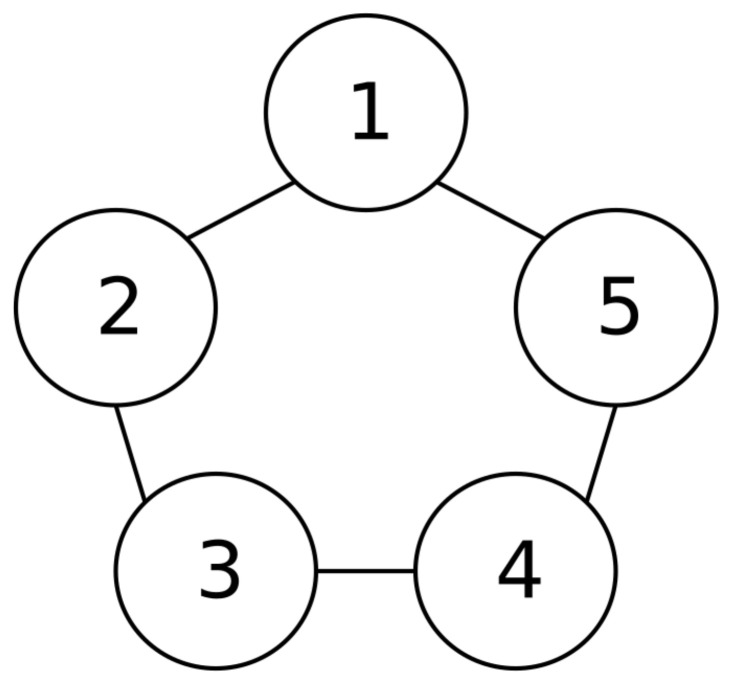
Topology of the communication graph.

Through this example, the MAS consisting of five agents is considered, whose topology of the communication graph as shown in Figure 2. The model is as follows:

(88) $$\left\{\begin{array}{cc} dx_{i,1} & =\left[x_{i,2}+h_{i,1}\left(X_{i,1}\right)\right]dt+F_{i,1}dw+\int_{R}G_{i,1}N\left(dt,d\zeta \right)\\ dx_{i,2} & =\left[u_{i}+h_{i,2}\left(X_{i,2}\right)\right]dt+F_{i,2}dw+\int_{R}G_{i,2}N\left(dt,d\zeta \right)\\ y_{i} & =x_{i,1} \end{array}\right.$$

where i=1,2,3,4,5, the Brownian motion term and Poisson jump term are $F=-\frac{\pi }{2}x_{1}^{2}$ and $G=\left(x_{2}-x_{1}\right)\zeta$, respectively, and the initial states are selected as $x_{1}\left(0\right)=\left[0.1,0.1\right]$, $x_{2}\left(0\right)=\left[0.2,0.2\right]$, $x_{3}\left(0\right)=\left[0.3,0.3\right]$, $x_{4}\left(0\right)=\left[0.4,0.4\right]$, and $x_{5}\left(0\right)=\left[0.5,0.5\right]$. The unknown functions in system (88) are:

$$\begin{array}{c} \begin{array}{cc} h_{1,1} & =h_{2,1}=h_{3,1}=h_{4,1}=h_{5,1}=0\\ h_{1,2} & =x_{1,1}-0.25x_{1,2}-x_{1,1}^{3}\\ h_{2,2} & =x_{2,1}-0.25x_{2,2}-x_{2,1}^{3}+0.1{\left(x_{2,1}^{2}+x_{2,2}^{2}\right)}^{1/2}\\ h_{3,2} & =x_{3,1}-0.25x_{3,2}-x_{3,1}^{3}+0.2{\left(x_{3,1}^{2}+2x_{3,2}^{2}\right)}^{1/2}\\ h_{4,2} & =x_{4,1}-0.25x_{4,2}-x_{4,1}^{3}+0.2{\left(2x_{4,1}^{2}+2x_{4,2}^{2}\right)}^{1/2}\\ h_{5,2} & =x_{5,1}-x_{5,2}+0.5{\left(x_{5,1}^{2}+x_{5,2}^{2}\right)}^{1/2} \end{array} \end{array}$$

Each of the five agents has the following local objective functions:

$$\begin{array}{c} \begin{array}{cc} \; & {£}_{1}\left(x_{1,1}\right)=x_{1,1}^{2}-2x_{1,1}+2\\ \; & {£}_{2}\left(x_{2,1}\right)=x_{2,1}^{2}-4x_{2,1}+6\\ \; & {£}_{3}\left(x_{3,1}\right)=x_{3,1}^{2}-6x_{3,1}+12\\ \; & {£}_{4}\left(x_{4,1}\right)=x_{4,1}^{2}-8x_{4,1}+20\\ \; & {£}_{5}\left(x_{5,1}\right)=x_{5,1}^{2}-10x_{5,1}+30 \end{array} \end{array}$$

Then, the penalty function is defined as (7), and the following conditions must be met to obtain the optimum solution for DOP:

$$\begin{array}{c} \begin{array}{c} \frac{\partial P(x_{1}^{*})}{\partial x_{1}^{*}}=0. \end{array} \end{array}$$

According to Equations (33), (36), (37) and (40)–(43), design the parameters update laws, the error compensation signal, the virtual control law and the control input as follows:

(89) $$\begin{array}{cc} a_{i,1} & =\frac{1}{d_{i}}(2m_{i}{\dot{b}}_{i}-c_{i,1}s_{i,1}-s_{i,1}+\sum_{j\in N_{i}}a_{ij}({\hat{x}}_{j,2}\\ & +\psi_{j,1}^{T}\varphi_{j,1}))-\psi_{i,1}^{T}\varphi_{i,1}\\ {\dot{\xi }}_{i,1} & =d_{i}\left(\xi_{i,2}+{\bar{a}}_{i,2}-a_{i,1}\right)-c_{i,1}\xi_{i,1}-\xi_{i,1}\\ {\dot{\psi }}_{i,1} & =r_{i,1}d_{i}\varphi_{i,1}z_{i,1}-{\bar{r}}_{i,1}\psi_{i,1}\\ {\dot{\psi }}_{j,1} & =-r_{j,1}\varphi_{j,1}z_{j,1}-{\bar{r}}_{j,1}\psi_{j,1}\\ u_{i} & ={\dot{\bar{a}}}_{i,2}-d_{i}s_{i,1}-c_{i,2}s_{i,2}-\frac{3}{2}s_{i,2}-\psi_{i,2}^{T}\varphi_{i,2}\left({\hat{X}}_{i,2}\right)\\ {\dot{\xi }}_{i,2} & =-d_{i}\xi_{i,1}-c_{i,2}\xi_{i,2}-\frac{3}{2}\xi_{i,2}\\ {\dot{\psi }}_{i,2} & =r_{i,2}\varphi_{i,2}z_{i,2}-{\bar{r}}_{i,2}\psi_{i,2} \end{array}$$

In addressing the entirety of the design parameters, $c_{i,1}$, $c_{i,2}$ stand out as pivotal to the system’s performance. Its direct correlation with the system’s convergence accuracy establishes it as the paramount tuning element. Concurrently, the configuration of neural network parameters $r_{i,1}$, $r_{i,2}$, $r_{j,1}$, ${\bar{r}}_{i,1}$, ${\bar{r}}_{i,2}$, and ${\bar{r}}_{j,1}$ merits equal attention, given their significant influence on the control inputs. Furthermore, the observer’s parameters $\iota_{1,1}$, $\iota_{2,1}$, $\iota_{3,1}$, $\iota_{4,1}$, $\iota_{5,1}$, $\iota_{1,2}$, $\iota_{2,2}$, $\iota_{3,2}$, $\iota_{4,2}$, and $\iota_{5,2}$ are equally essential, serving as the critical intermediate variables within the framework of the virtual control law.

Regarding the control parameters $c_{i,1}$, $c_{i,2}$, they exert a direct influence on the system’s control input. While higher values can enhance the rate of convergence, excessively high values might result in overly large control inputs, which could negatively impact the system’s overall performance. Consequently, we have selected moderate values for $c_{i,1}$ and $c_{i,2}$ to ensure a swift and stable response from the system. The parameters are chosen as $c_{i,1}=4$, $c_{i,2}=3$.

When adjusting the adaptive law parameters $r_{i,1}$, $r_{i,2}$, and $r_{j,1}$, we found that increasing the values of $r_{i,1}$, $r_{i,2}$, and $r_{j,1}$ can amplify the system’s output jitter, while decreasing them might prevent the neural network from outputting effectively and adapting to changes in nonlinear functions. Therefore, we selected moderate values $r_{i,1}=r_{i,2}=r_{j,1}=1$ to balance the system’s stability with the neural network’s responsiveness. Additionally, increasing the values of ${\bar{r}}_{i,1}$, ${\bar{r}}_{i,2}$, and ${\bar{r}}_{j,1}$ may speed up convergence, but excessively high values could cause the neural network to converge directly to zero, thereby losing functionality. Therefore, we have chosen an appropriate value for ${\bar{r}}_{i,1}$, and ${\bar{r}}_{i,2}$, ${\bar{r}}_{j,1}$ to ensure that the neural network can operate effectively. Here, ${\bar{r}}_{i,1}={\bar{r}}_{i,2}={\bar{r}}_{j,1}=80$.

With regard to the observer parameters, we recognize that an increase in these parameters can reduce the observer error. However, we must take into account the constraints of practical applications, so we cannot increase these parameters indefinitely. We have chosen an appropriate value to ensure the performance of the observer and the stability of the system. So the design parameters for the observer are selected as $\iota_{1,1}=\iota_{2,1}=\iota_{3,1}=\iota_{4,1}=\iota_{5,1}=5$, $\iota_{1,2}=\iota_{2,2}=\iota_{3,2}=\iota_{4,2}=\iota_{5,2}=15$ and the initial states are designed as ${\hat{x}}_{1}=\left[0.2,0.2\right]$, ${\hat{x}}_{2}=\left[0.3,0.3\right]$, ${\hat{x}}_{3}=\left[0.4,0.4\right]$, ${\hat{x}}_{4}=\left[0.5,0.5\right]$, and ${\hat{x}}_{5}=\left[0.6,0.6\right]$.

**Figure 3:**
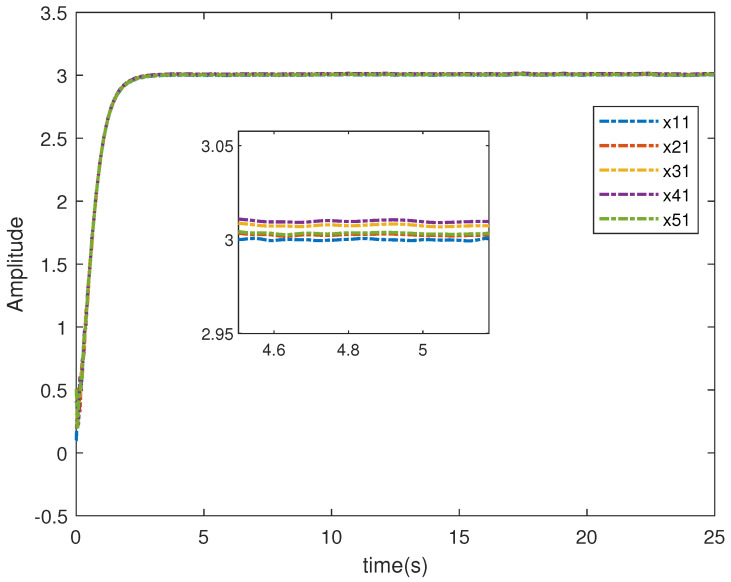
The system state $x_{i,1}\left(i=1,\cdots ,5\right)$.

In this example, we give the first agent $b_{1}=1$, the second agent $b_{2}=2$, the third agent $b_{3}=3$, the fourth agent $b_{4}=4$, and the fifth agent $b_{5}=5$. Through calculation, we can see that the optimal value of the five agents is $x_{i,1}^{*}=3$. From the Figure 3, there is evidence that the five agents will eventually converge to the optimal value.

**Figure 4:**
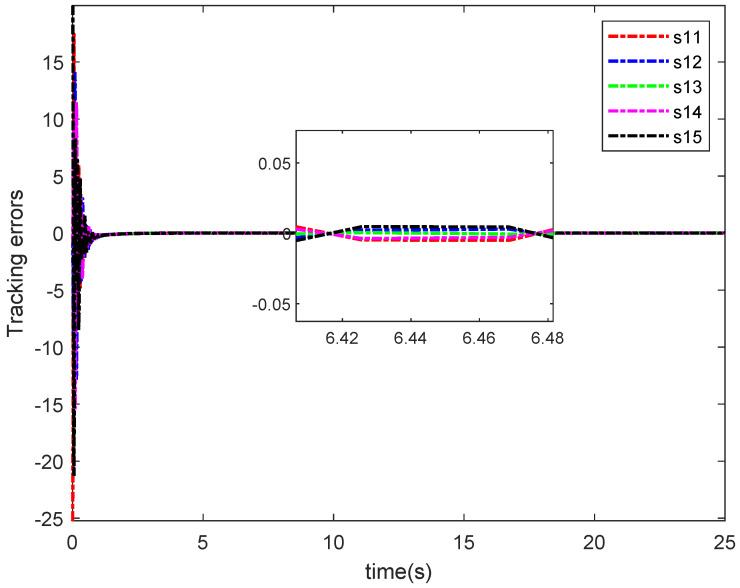
The error $s_{i,1}\left(i=1,\cdots ,5\right)$.

**Figure 5:**
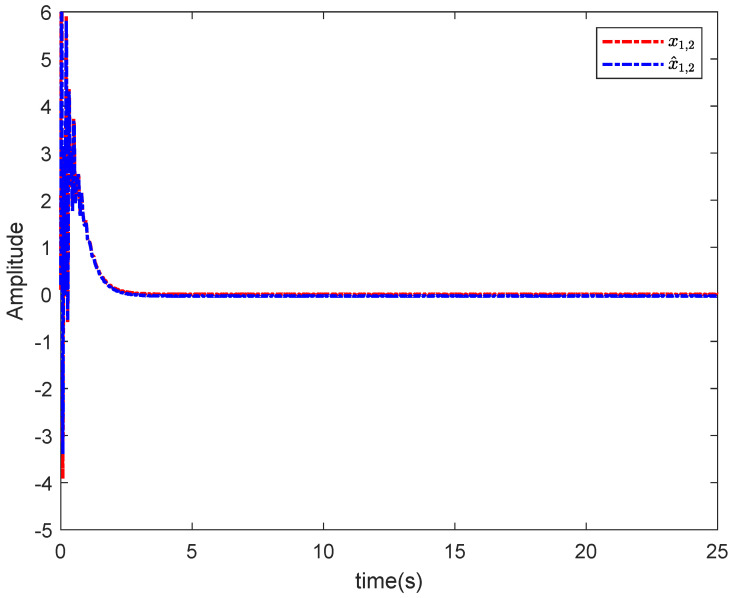
$x_{1,2}$ and its estimation.

**Figure 6:**
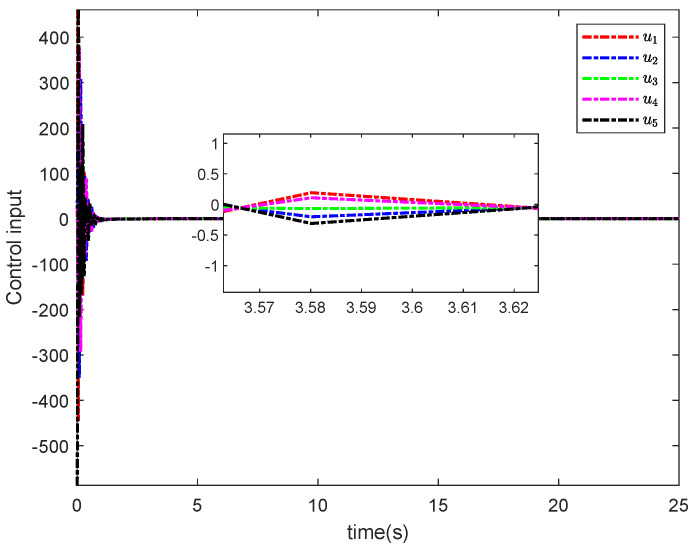
Control input $u_{i}$.

**Figure 7:**
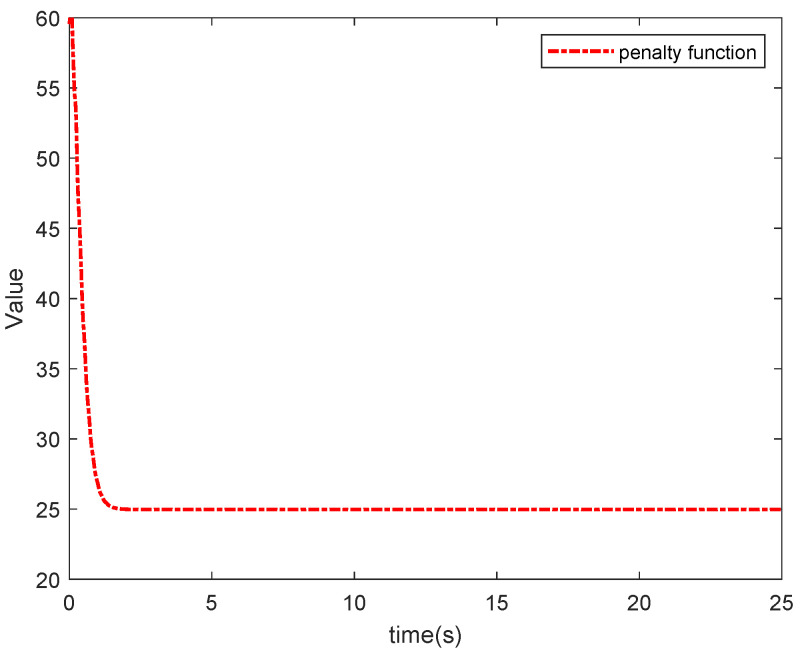
The value of the penalty function.

**Figure 8:**
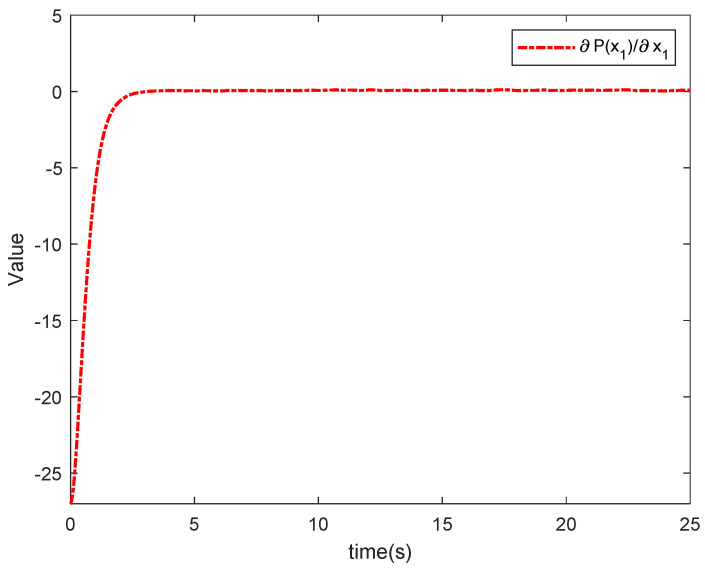
The value of the gradient.

The simulation results in this simulation are shown in Figure 3, Figure 4, Figure 5, Figure 6, Figure 7 and Figure 8. Figure 3 indicates that the output of each agent are consistent with the optimal solution, and a certain extent of error in the figure. Figure 4 shows the tracking error trajectories $s_{i,1}$, which clearly shows that $s_{i,1}$ converge to zero quickly. Through Figure 5, we put the output of the agent 1 as an example to make a comparison between the true and estimated values. The control input $u_{i}$ is presented in Figure 6. Figure 7 displays the value of the penalty function, and we may conclude that the proposed control protocol will minimize the penalty function. Figure 8 displays the value of gradient, which can be clearly established the gradient value is converging well towards zero.

Under the above simulation results, the proposed algorithm will guarantee that all agents reach the optimal solution in the MASs with dynamic uncertainty and stochastic noise. Tracking errors are converging towards a tiny region of source in a short period of time, and all agents eventually tend to optimize. Controllers designed with this approach not only make sure that all agents provide excellent tracking performance for the system containing nonlinear uncertainties and random noise, it also considers the DOP. At the same time, the value of the penalty function successfully trades down to the minimal value.

## 5. Conclusions

This paper studies the DOP for high-order MASs with nonlinear functions and Lévy stochastic noise. The penalty function is built using the properties of the undirected communication graph and GOF to ensure that all agents achieve the optimal value of DOP while reaching consensus. To avoid “complexity explosion”, we take the command-filtered into account to design the adaptive NNs backstepping control, and the error compensation mechanism is applied to remove the influence of the filtering errors. The stability of the system is analyzed by combining the generalized Itô’s formula with the Lyapunov function method. Simulation results demonstrate that the developed algorithm can make the outputs of all agents reach the optimal value with bounded errors.

This study holds significant implications in practical application fields. Compared the research in to [9], which is confined to the analysis of master–slave systems involving only two agents, this paper expands the scope of research by applying stochastic systems to MASs and successfully addressing the DOP, enabling the research outcomes to be applied to a more diverse range of practical scenarios. However, this paper does not cover MASs with full-state constraints, as discussed in [46]. To further deepen the research, this paper plans to draw on the research methods of [47] and adopt an innovative Policy Iteration (PI) algorithm to explore the online adaptive optimal control problem of nonlinear multi-agent systems.

## Data Availability

The original contributions presented in the study are included in the article, further inquiries can be directed to the corresponding author.
